# A pipeline approach to single-particle processing in *RELION*

**DOI:** 10.1107/S2059798316019276

**Published:** 2017-04-20

**Authors:** Rafael Fernandez-Leiro, Sjors H. W. Scheres

**Affiliations:** a MRC Laboratory of Molecular Biology, Francis Crick Avenue, Cambridge Biomedical Campus, Cambridge CB2 0QH, England

**Keywords:** *RELION*, cryo-EM, single-particle analysis

## Abstract

The formal concept of a workflow to single-particle analysis of cryo-electron microscopy (cryo-EM) images in the *RELION* program is described. In this approach, the structure-determination process is considered as a graph, where intermediate results in the form of images or metadata are the vertices, and different functionalities of the program are the edges. The new implementation automatically logs all user actions, facilitates file management and disk cleaning, and allows convenient browsing of the history of a project. Moreover, new functionality to iteratively execute consecutive jobs allows on-the-fly image processing, which will lead to more efficient data acquisition by providing faster feedback on data quality. The possibility of exchanging data-processing procedures among users will contribute to the development of standardized image-processing procedures, and hence increase accessibility for new users in this rapidly expanding field.

## Abstract 2

The formal description of a workflow to cryo-EM structure determination in the *RELION* program allows standardization of procedures and on-the-fly image processing during data acquisition.

## 1. Introduction   

Recent advances in cryo-EM instrumentation and image-processing software have substantially expanded the scope of structure determination by single-particle analysis (Fernandez-Leiro & Scheres, 2016 ▸). As a result, the field is growing rapidly. With many new users turning to cryo-EM structure determination, efforts in methods development are increasingly focused on improving the accessibility of the technique. Examples of this are the development of robots for sample preparation and transfer of the sample into the microscope (Cheng *et al.*, 2007 ▸; Vos *et al.*, 2008 ▸; Kim *et al.*, 2010 ▸; Coudray *et al.*, 2011 ▸); the introduction of automated data-acquisition and processing software (Suloway *et al.*, 2005 ▸; Stagg *et al.*, 2006 ▸; Mastronarde, 2005 ▸; Li *et al.*, 2015 ▸); the introduction of image-processing software suites with convenient graphical user interfaces (GUIs; Tang *et al.*, 2007 ▸; Hohn *et al.*, 2007 ▸; Baxter *et al.*, 2007 ▸; de la Rosa-Trevín *et al.*, 2013 ▸); and the development of integrated software environments and file formats that allow convenient interchanging between the different programs (Lander *et al.*, 2009 ▸; de la Rosa-Trevín *et al.*, 2016 ▸; Marabini *et al.*, 2016 ▸). It is interesting to note that a similar development towards automation and ease of use happened in the related field of macromolecular structure determination by X-ray crystallography during the 1990s and 2000s (Blundell *et al.*, 2002 ▸).

This paper describes the implementation of a pipeline approach to cryo-EM structure-determination protocols in the *RELION* program (Scheres, 2012*b* ▸). *RELION* is based around an empirical Bayesian approach to single-particle analysis (Scheres, 2012*a* ▸), where important parameters that describe the signal-to-noise ratios of the data are inferred from the data themselves. This reduces the need for expertise to run the program, and makes it intrinsically suitable for automation. Since its introduction in 2012, a convenient GUI has further enriched the ease of use of *RELION*, but until now the concept of a workflow did not exist explicitly within the program. To facilitate the generation of standardized and (semi-)automated procedures for structure determination, we describe here the formal description of a workflow, which we call a pipeline, in the latest *RELION* release (v.2.0).

## 2. Implementation of the pipeline   

### 2.1. Jobs and nodes   

The process of cryo-EM structure determination can be described as a directed acyclic graph, consisting of vertices and edges. We will refer to the vertices as *nodes*, and a total of 14 different types of nodes, representing different forms of data or metadata, have been defined in* RELION*-2.0 (Table 1 ▸). Depending on the type, nodes are stored on the computer disk in the form of STAR (Hall, 1991 ▸), PDF, MRC (Cheng *et al.*, 2015 ▸) or plain-text files. The edges of the graph represent processes that act on these data. The edges are called *jobs*, and a total of 18 different types of job have been implemented (Table 2 ▸). With the exception of the ‘Import’ job, all job types take one or more nodes as input and produce one or more nodes as output. Different job types take different types of input and output nodes (see Table 2 ▸). Using output nodes from one job as input for the next job builds up a graph, which we refer to as the *pipeline*.

**Figure 1:**
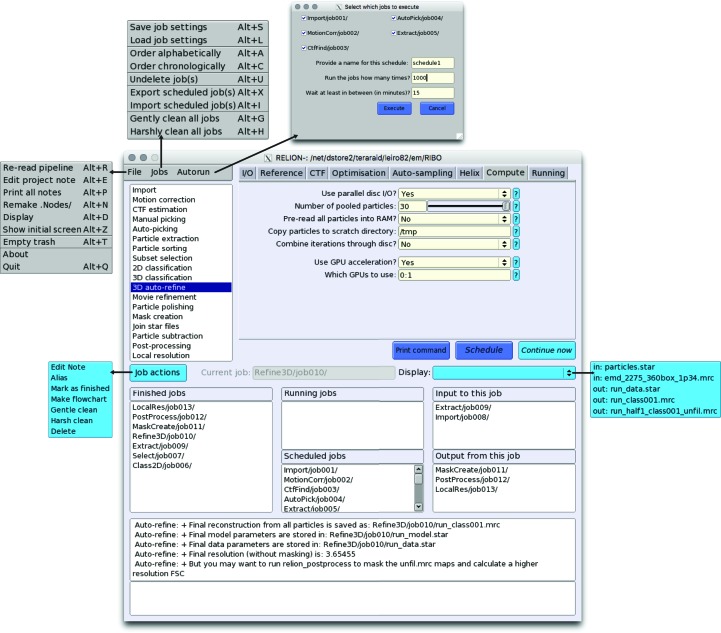
The *RELION*-2.0 GUI.

The 18 job types have all been implemented in a new GUI (Fig. 1 ▸), which aims to encapsulate the entire functionality that is needed to perform cryo-EM structure determination. One can start with the import of a set of two-dimensional micrographs or movies, and proceed from there. (However, *de novo* generation of a three-dimensional reference model from the experimental images has not been implemented, and three-dimensional references generated in other programs need to be imported into the pipeline.) New jobs can be selected from the job-type list on the top left of the GUI. Selecting a job type from this list will load the corresponding parameter input fields on the top right part of the GUI. In order to reduce the risk of selecting incorrect input files, the fields that correspond to input nodes have ‘Browse’ buttons that will only display nodes with the expected types that already exist in the pipeline. Internally, the node-type-specific browse buttons use a hidden directory called Nodes/. Sometimes this directory becomes corrupted, in which case it can be re-generated using the ‘Remake.Nodes/’ entry from the File menu on the GUI.

### 2.2. Job execution   

When all parameters of a job have been selected, a job can be executed using the ‘Run now!’ button on the GUI. All jobs that are executed through the GUI will be added to the pipeline, which is stored as a STAR file on the computer disk. This STAR file contains lists of all nodes and all processes in the pipeline, as well as lists of which nodes are used as input or output for which jobs. All jobs within a pipeline are numbered consecutively, and a new directory is created inside the project directory for every new job that is executed. These directories are nested inside a higher directory that reflects the job type. For example, if a two-dimensional (2D) classification job is the tenth job to be executed in the pipeline, then its output will be stored in a directory called Class2D/job010/. (See Table 2 ▸ for the higher directory names for all job types.) To facilitate the recognition of jobs by the user, the concept of a job alias has also been implemented. An alias is a unique name, a text string that does not contain special characters such as spaces or slashes, that provides a more intuitive description of a job. The creation of an alias for a job leads to the generation of a symbolic link to that job’s output directory on the computer disk.

Upon execution of the job, its name (either its output directory or its alias) is added to a list of ‘Running jobs’ on the GUI, and it will remain there until the files of all output nodes have been detected on the computer disk. When the files of all expected output nodes are present, the status of the job will change from running to finished, and on the GUI the job will move to the list of ‘Finished jobs’. When a job is executed, the GUI also creates a text file called note.txt inside the job’s output directory, which will contain a time stamp when the job was executed and the exact command-line arguments of the program used. The GUI will also open a text-editor window that allows the user to further comment on the intentions or characteristics of the job. The latter could serve as an electronic logbook for the user.

Some jobs are stopped before they reach their intended result. In order to finish such jobs, the ‘Run now!’ button on the GUI changes to a ‘Continue now’ button when a job is selected from any of the lists in the lower part of the GUI. Continuing a job works in different manners for different types of jobs. Pre-processing jobs such as ‘Motion correction’, ‘CTF estimation’, ‘Manual picking’, ‘Auto-picking’ and ‘Particle extraction’, as well as ‘Movie refinement’, will skip any micrographs for which the expected output files are already on the computer disk. The likelihood-optimization jobs ‘2D classification’, ‘3D classification’ and ‘3D auto-refine’ can be continued from their last finished iteration by providing the corresponding _optimiser.star file. Sometimes, it is not necessary to continue a job that was stopped prematurely. In this case, the user can also use the ‘Mark as finished’ option under the ‘Job action’ button, which will move the job from the ‘Running jobs’ to the ‘Finished jobs’ lists. In the case of a likelihood-optimization job, the expected output nodes in the pipeline will then be replaced for the output files of the last finished iteration.

It is important to note that *RELION* does not retain control over the running job: it will merely wait for the expected output files to appear on the computer disk. This means that there is no functionality for the user to kill a submitted job through the GUI or to track its job ID, which should instead be performed through the operating system or the job queue.

### 2.3. Browsing the project history   

The user can conveniently browse through all jobs in the pipeline by clicking jobs in the lists of ‘Finished jobs’, ‘Running jobs’ or ‘Scheduled jobs’. When the user clicks on a job in these lists, the parameters of that job are read from a file called run.job inside that job’s output directory, and these are loaded into the parameter input fields on the top part of the GUI. Moreover, any (upstream) jobs that generated the input nodes for the selected job are shown on the GUI in the list called ‘Input to this job’, and any (downstream) jobs that take the output nodes of this job as input are shown in the list called ‘Output from this job’. Clicking on jobs in the latter two lists allows convenient browsing back and forth through the history of the project. In addition, the user can explore how jobs are connected to each other by generating vector-based, and thereby conveniently editable, flowcharts in PDF format.

### 2.4. Analysing results   

For each job in the pipeline, the ‘Display’ button on the GUI allows visualization of its own input and output nodes. This guides the user by presenting only a few options of which files to display for each job. For several job types, it is often worthwhile to also inspect part of the intermediate results. For this purpose, some job types will output a file called logfile.pdf, which presents the most relevant necessary intermediate results in a convenient, graphical manner. Examples include the movement tracks of individual movies from ‘Motion correction’ jobs, the *B*-factor plots and per-particle movement tracks from ‘Particle polishing’, and FSC as well as Guinier plots from ‘Post-processing’ jobs.

A more general display functionality that allows visualization of any image, reminiscent of the ‘Display’ button in previous versions of *RELION*, is still available through the ‘File’ menu.

### 2.5. Disk management   

In a typical structure-determination project, pipelines can quickly become complicated as many different ways to process their data are explored. This may generate large amounts of intermediate results that occupy substantial amounts of space on the computer disk. The strict organization of output files in a separate directory for each job facilitates file management. The GUI implements functionality to delete jobs that are deemed to be no longer necessary by removing their output directories from the project directory. In addition, the user can also choose to clean output directories of jobs, in which case only intermediate files are removed but files necessary for the pipeline are retained. Both functionalities are accessible through the ‘Job actions’ button. To protect the user from unwanted loss of data, upon deletion or cleaning of a job all output files are initially moved to a Trash/ folder inside the project directory. Entire jobs can be ‘undeleted’ from the ‘Jobs’ menu, while specific files can also be recovered manually using the Linux command line. To free disk space, files can be removed permanently through an ‘Empty Trash’ option on the ‘File’ menu.

## 3. On-the-fly processing and exchanging procedures   

Instead of immediately executing a job once all its parameters have been selected, the user can also use the ‘Schedule’ button to schedule a job for execution at a later stage. Even though the files for the output nodes of scheduled jobs do not exist yet, they will still be added to the .Nodes/ directory that is used by the ‘Browse’ buttons on the parameter input fields. Thereby, once a job is scheduled one can select its expected output nodes as input for another job, which can then also be scheduled. In this manner, one can schedule multiple, consecutive jobs for future execution.

Execution of scheduled jobs can be performed through the ‘Autorun’ menu on the GUI. This will launch a separate window in which the user can select which of the scheduled jobs to execute. In addition, the user can opt to cycle through the selected scheduled jobs multiple times and specify a minimum time between subsequent iterations. Combined with the functionality to continue jobs explained above, this provides a simple mechanism for on-the-fly processing of data during acquisition. For example, one could schedule an ‘Import’ job that imports all movie files in a given directory, followed by ‘Motion correction’, ‘CTF estimation’, ‘Auto-picking’ and ‘Particle extraction’ jobs. This cycle of consecutive jobs could be repeated many times while the data are being acquired. In each iteration, the jobs will only act on those movies that have not been processed before.

Once enough particles have been extracted, the output from the ‘Particle extraction’ job above may also be used as input for a ‘2D classification’ job. This will lead to the calculation of reference-free two-dimensional class averages, which typically provide useful insights into the quality of the data. Probably one would not want to perform the (computationally more expensive) two-dimensional classification job as often as one would want to pre-process the incoming movies. Therefore, multiple executions of scheduled jobs can be run independently. For example, one could pre-process new micrographs from ‘Import’ to ‘Particle extraction’ every 5 min, but only execute ‘2D classification’ with the extracted particles every hour. Provided sufficient computer resources are at hand to process the incoming data, this procedure allows the user to assess data quality from the inspection of two-dimensional class averages during data acquisition. Thereby, on-the-fly data processing will allow the user to change their data-acquisition scheme in case the data are unsatisfactory. Even ‘3D classification’ or ‘3D auto-refine’ jobs may be included in on-the-fly processing. This may be particularly useful to assess whether cofactors are bound to complexes, or whether the acquired data are capable of reaching high resolution. It is also possible to change the parameters of jobs that are scheduled by clicking on the job in the scheduled jobs list, modifying its entry fields and using the ‘Save job settings’ options in the ‘Jobs’ menu. This could be useful, for example, in the iterative execution of two-dimensional or three-dimensional classification where it becomes clear that too low a number of classes was used initially.

The capability of executing multiple, previously scheduled jobs is also relevant for developing standardized procedures for image processing. To facilitate this, scheduled jobs can be exported from the pipeline using the corresponding option from the ‘Jobs’ menu. Exporting scheduled jobs will change the directory structure with the numbered jobs from the current pipeline to a more generic directory structure. This generic structure can then be copied into the directory of a different project, where it can be imported into the pipeline by again using the corresponding option from the ‘Jobs’ menu. This will allow different users to share their image-processing procedures, which will be of help for inexperienced users and may further facilitate the development of automated and standardized procedures.

## 4. Results   

### 4.1. Test-case description   

To demonstrate the new pipeline, we reprocessed a data set from the EMPIAR database (Iudin *et al.*, 2016 ▸): entry 10028 (Wong *et al.*, 2014 ▸). This cryo-EM data set comprises 1081 16-frame movies of 4096 × 4096 pixels that were collected using a Falcon-II direct-electron detector on an FEI Polara microscope. The sample contained a mixture of (cytoplasmic) 80S ribosomes (at 160 n*M*) that were purified from *Plasmodium falciparum* parasites and a 1 m*M* solution of the antiprotozoan drug emetine. In the original study, the structure was solved to an overall resolution of 3.2 Å using a beta version of *RELION*-1.3.

All experiments described below were performed on a single desktop machine equipped with a Intel i7-6800K 3.4 GHz six-core CPU, 64 GB of RAM and two Titan-X (Pascal) GPUs, which was recently acquired for less than £3000. GPU acceleration was used for motion correction in *MotionCor*2 (Zheng *et al.*, 2016 ▸) and for CTF parameter estimation in *Gctf* (Zhang, 2016 ▸), as well as for auto-picking, classification and refinement in *RELION*-2.0 (Kimanius *et al.*, 2016 ▸).

### 4.2. Simulated on-the-fly processing   

In an attempt to simulate the data-acquisition process, we copied a single movie per minute into a micrographs directory. The copying process was started at 14:00 in the afternoon and continued throughout the night until 08:30 the morning after. Although this is admittedly an oversimplification of the data-acquisition process (for example, the data set only contains high-quality images, did not stall *etc.*), our simulation still illustrates the functionality of on-the-fly data processing, which is already in active use at our cryo-EM facility.

To perform on-the-fly data processing, we scheduled consecutive jobs to (i) import all movies in the micrographs directory, (ii) run *MotionCor*2, (iii) run *Gctf*, (iv) run auto-picking and (v) extract the particles. The scheduled jobs were then executed in a continuous loop, with a minimum of 1 min between subsequent iterations, during the entire copying process. Motion correction was performed in five patches and included dose-weighting. *Gctf* estimation used equiphase averaging to increase the signal-to-noise ratios in the Thon-ring images. Auto-picking (Scheres, 2015 ▸) was performed with a single Gaussian blob as a template. The latter is a new option in *RELION*-2.0 and involves the choice of two parameters: the width and the height of the Gaussian. The width of the Gaussian was chosen to be similar to the expected diameter of the particles (270 Å). The height of the Gaussian is affected by the signal-to-noise ratio in the images, and a suitable value (0.3) was determined by a test run on the first few micrographs. Threefold downscaled particles were initially extracted in boxes of 120 × 120 pixels. The two GPUs on our machine were used to process two movies in parallel, if needed. The pre-processing procedure took approximately 1 min per movie, half of which was taken by *MotionCor*2. Consequently, data processing could be performed as fast as the data were coming in, and the entire pre-processing finished only 4 min after the last movie was copied.

**Figure 2:**
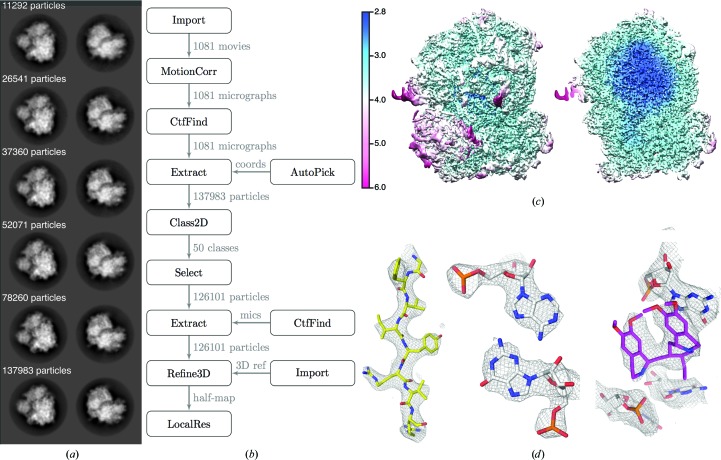
Results for the EMPIAR-10028 data set. (*a*) Two representative reference-free two-dimensional class averages at different times during the data-copying process. (*b*) Automatically generated flowchart of the data-processing process. (*c*) Front view and slice through a map that is filtered according to the local resolution as estimated by *RELION*. (*d*) Close-up views of details in the map (a β-strand, an RNA base pair and the emetine compound).

After 111 movies had been copied, a second independent loop of ‘2D classification’ jobs was also executed, with a minimum of 1 h between subsequent iterations. These jobs generated reference-free two-dimensional class averages for all of the extracted particles that had been acquired thus far. These jobs competed for the same two GPUs as were also used by the pre-processing jobs. The execution times for the two-dimensional classification jobs gradually increased as more particles were extracted from the available micrographs. The first job took only 50 min, whereas the last job took 3.5 h and finished at 12:30 the next day, *i.e.* 4 h after the data copying ended. Fig. 2 ▸(*a*) shows how the class averages developed throughout the copying process. Excellent two-dimensional class averages, showing different projections of 80S ribosomes with many protein and RNA-like features, confirmed the high quality of the data set during the early stages of copying.

Subsequently, by manually inspecting the final two-dimensional class averages, we selected 126 101 particles, re-extracted these in boxes of 360 × 360 pixels (without downscaling) and used these as input for a ‘3D auto-refine’ job. We used the same initial three-dimensional reference (EMDB-2275; Bai *et al.*, 2013 ▸) as was used in the original study (Wong *et al.*, 2014 ▸). The refinement was followed by standard ‘Mask creation’ and ‘Post-processing’ jobs. Finally, to estimate the variations in local resolution throughout the reconstruction, we used a new feature in the ‘Local resolution’ job of *RELION*-2.0. In this feature, we use phase randomization (Chen *et al.*, 2013 ▸) to correct for the solvent effects of a small, spherical mask, which is slided over the entire map. In total, three-dimensional auto-refine, post-processing and local resolution estimation took 16 h on our desktop machine. Fig. 2 ▸(*b*) shows the automatically generated flowchart of the data-processing pipeline, Fig. 2 ▸(*c*) shows the local resolution estimates and Fig. 2 ▸(*d*) shows details of the reconstructed density on top of the original atomic model (Wong *et al.*, 2014 ▸).

### 4.3. Standardization and procedure exchange   

To illustrate the functionality to exchange data-processing procedures among users, we exported the entire pipeline for the re-processing of the EMPIAR-10028 data set, as described above, and provide a zip archive as Supporting Information to this paper. Users interested in adopting or adapting this procedure can uncompress this archive in their project directory and import the file called ./export1/exported.star through the corresponding option on the ‘Jobs’ menu.

## 5. Conclusion   

We describe how a pipelined approach to cryo-EM structure determination formalizes the concept of a directed acyclic graph in *RELION*-2.0. The new approach improves the user experience by reducing the scope for errors, automating bookkeeping, standardizing the organization of output files, allowing straightforward browsing through the project’s history and providing convenient disk-cleaning functionalities. The functionality to schedule multiple, consecutive jobs for future execution, and the option to execute these jobs in iterative loops, allows on-the-fly processing of the data while they are being acquired. This will lead to more efficient use of microscopy time, as more informed assessments of data quality can be made while the data are still being acquired. Finally, the possibility of exporting and importing (parts of) pipelines will allow the exchange of data-processing strategies between users, which will further improve the accessibility of the technique to inexperienced users and facilitate the development of standardized data-processing procedures. *RELION*-2.0 is open-source software. For download instructions and documentation, please refer to the *RELION* Wiki pages at http://www2.mrc-lmb.cam.ac.uk/relion. For user questions and feedback, please use the CCPEM mailing list at http://www.jiscmail.ac.uk/CCPEM.

## Supplementary Material

Supporting Information.. DOI: 10.1107/S2059798316019276/ic5096sup1.zip

## Figures and Tables

**Table 1:** Node types in *RELION*-2.0

ID	Node type	Description
0	Movies	A STAR file with two-dimensional movies and their metadata
1	Micrographs	A STAR file with two-dimensional micrographs and their metadata
2	Particle coordinates	A text file with the coordinate-file suffix
3	Particles	A STAR file with individual particles and their metadata
4	Movie particles	A STAR file with particle movie frames and their metadata
5	2D references	A STAR file or MRC stack with two-dimensional (reference) images
6	3D reference	A three-dimensional reference map (in .MRC format)
7	3D mask	A three-dimensional mask (in .MRC format)
8	Model	A _model.star file for selecting classes
9	Optimiser	An _optimiser.star file for continuing optimizations
10	Half-map	One of two unfiltered half-maps from an auto-refinement
11	Final map	A post-processed map
12	ResMap	A map with local resolution estimates
13	Logfile	A PDF file with additional output from a process

**Table 2:** Job types in *RELION*-2.0

ID	Job type	Input nodes	Output nodes	Output directory	Description
0	Import	—	0–7, 10	Import/	Import files into the pipeline
1	Motion correction	0	0, 1, 13	MotionCorr/	Wraps to external motion-correction programs
2	CtfFind	1	1	CtfFind/	Wraps to external CTF estimation programs
3	Manual picking	1	1, 2	ManualPick/	Manual picking of particles
4	Auto-picking	1, 5	2	AutoPick/	Automated picking of particles
5	Particle extraction	1–3	3	Extract/	Extraction of particle boxes from the micrographs
6	Particle sorting	3, 5, 8	3	Sort/	Sort particles based on statistics in differences from reference images
7	Subset selection	1–3, 8	1, 3, 5	Select/	Select classes or subsets from lists of micrographs or particles
8	2D classification	3, 9	3, 8, 9	Class2D/	Reference-free two-dimensional class averaging
9	3D classification	3, 6, 7, 9	3, 6, 8, 9	Class3D/	Unsupervised three-dimensional classification
10	3D auto-refine	3, 6, 7, 9	3, 6, 8–10	Refine3D/	Gold-standard three-dimensional auto-refinement
11	Particle polishing	0, 7	3, 11, 13	Polish/	Per-particle motion correction and radiation-damage weighting
12	Mask creation	6	7	MaskCreate/	Generate mask from binarized map
13	Join STAR files	0, 1, 3	0, 1, 3	JoinStar/	Join sets of particles, micrographs or movies
14	Particle subtraction	3, 6, 7	3	Subtract/	Subtract projections of (masked) maps from experimental particles
15	Post-processing	10, 7	11, 13	PostProcess/	Solvent-corrected FSC calculation and map sharpening
16	Local resolution	7, 10	12	ResMap/	Local resolution estimation
17	Movie refinement	4, 9	4, 6, 8, 10, 13	MovieRefine/	Extract particles from movies and align against three-dimensional reference
